# Metastatic Gastric Signet Ring Cell Adenocarcinoma Masquerading as Low Back Pain

**DOI:** 10.7759/cureus.11297

**Published:** 2020-11-02

**Authors:** Sarah Burroughs, Robert Post, Edward James

**Affiliations:** 1 Internal Medicine, Advocate Lutheran General Hospital, Park Ridge, USA; 2 Pathology, University of Illinois at Chicago, Chicago, USA; 3 Oncology, Advocate Lutheran General Hospital, Park Ridge, USA

**Keywords:** gastric cancer, signet ring cell adenocarcinoma, bone marrow infiltration, bone marrow metastasis

## Abstract

This case report describes a 53-year-old Hispanic male who initially presented with acute hip pain. During workup, sclerotic bone lesions of the lumbar spine were identified on computed tomography (CT) in addition to extensive adenopathy involving the chest and abdomen. Upper endoscopy revealed chronic active gastritis, however, biopsies were negative for malignancy. Eventual bone marrow biopsy showed extensive infiltration by sheets of malignant epithelial cells with signet ring cell formation. Not only is this case significant for maintaining a broad differential in patients presenting with bone pain, but it also demonstrates a diagnosis of metastatic signet ring cell adenocarcinoma confirmed on bone marrow biopsy, which was not detected via earlier endoscopy.

## Introduction

Gastric cancer is one of the most common cancers worldwide and the leading cause of death until the 1980s. At the time of diagnosis, 50% of gastric adenocarcinomas are metastatic. The most common metastatic distribution is the liver, peritoneum, and lymph nodes; less commonly, the bone, pulmonary system, central nervous system, and ovaries. The most common presenting symptoms at initial diagnosis are weight loss, early satiety, and epigastric abdominal pain [1]. The presentation of metastatic signet ring cell gastric cancer as bone pain without these preceding symptoms is rare. Tissue diagnosis to confirm gastric cancer is best obtained by upper gastrointestinal endoscopy with biopsies of any suspicious-appearing gastric ulceration or mass. This case highlights the importance of establishing a wide differential when approaching a patient with bone pain, as well as demonstrating the diagnosis of a malignancy via bone marrow biopsy, not otherwise diagnosed on biopsy of the primary site.

## Case presentation

**Figure 1:**
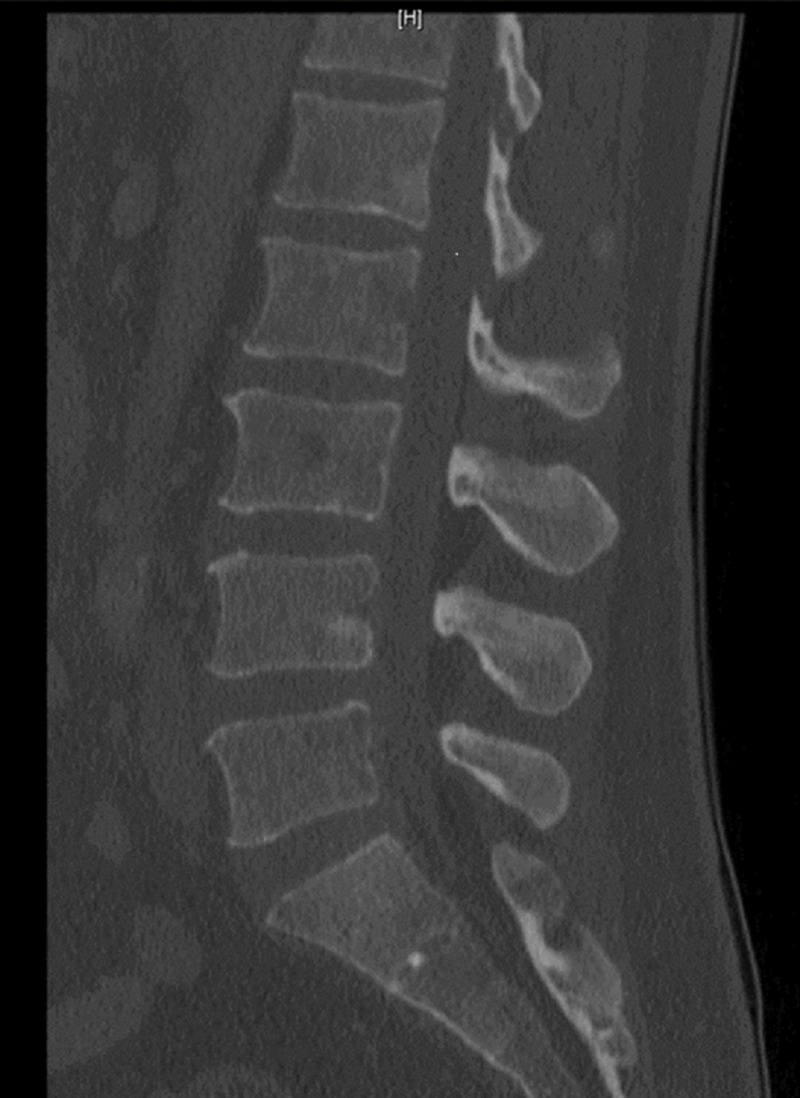
Sagittal view of CT lumbar spine revealing multiple small, nonspecific, round, radiolucent, and sclerotic bone lesions

A 53-year-old Hispanic male, with a history of diabetes, presented to the emergency department with acute lower back and hip pain of three weeks duration after lifting heavy equipment at work. He had been taking over-the-counter analgesics, with suboptimal relief. Other complaints included mild abdominal pain after analgesics, however, he reported no associated fevers, night sweats, weight loss, nausea, vomiting, hematochezia, or hematemesis. Physical examination was pertinent for a pale-appearing male, mild diffuse abdominal tenderness to palpation, and hip discomfort on palpation bilaterally. Laboratory work revealed microcytic anemia with a hemoglobin of 8.3 gm/dL (normal range 13.0-17.0 GM/DL) with an mean corpuscular volume (MCV) of 75, platelet count of 120 thousand/mcL (normal range 140-450 thousand/MCL), aspartate aminotransferase 95 unit/L (normal <38), alanine aminotransferase 49 unit/L (normal range <79), alkaline phosphatase 935 unit/L (normal range 45-117), total bilirubin 1.1 MG/DL (normal 0.2 - 1.0 MG/DL), and direct bilirubin 0.4. Calcium was within the normal range. Computed tomography (CT) of the lumbar spine revealed multiple small, radiolucent, and sclerotic bone lesions (Figure 1). CT of the chest, abdomen, and pelvis showed extensive adenopathy throughout the chest and abdomen and right middle lobe pulmonary nodule. A follow-up whole-body bone scan revealed increased radiotracer activity in the left proximal humerus, bilateral ribs, various levels of the thoracic and lumbar spine, sternum, left superior acetabulum, and right ischial spine suspicious for skeletal metastatic disease. Given anemia and suspected malignancy, the patient underwent an upper endoscopy, which revealed gastritis, but was otherwise unremarkable. Biopsies were negative for Helicobacter (H.) pylori and malignancy.

**Figure 2:**
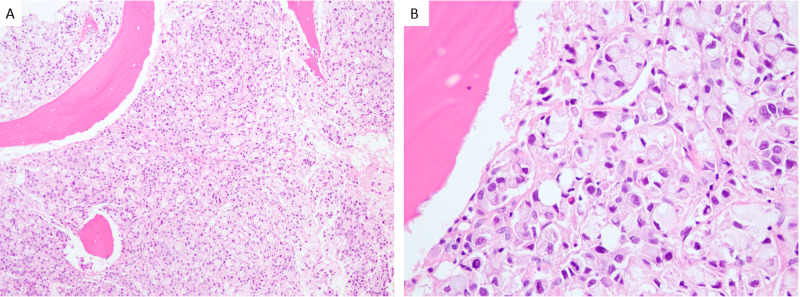
Bone marrow biopsy under 20x magnification (A) and 60x magnification (B) showing extensive infiltration by sheets of malignant epithelial cells with signet ring formation

**Figure 3:**
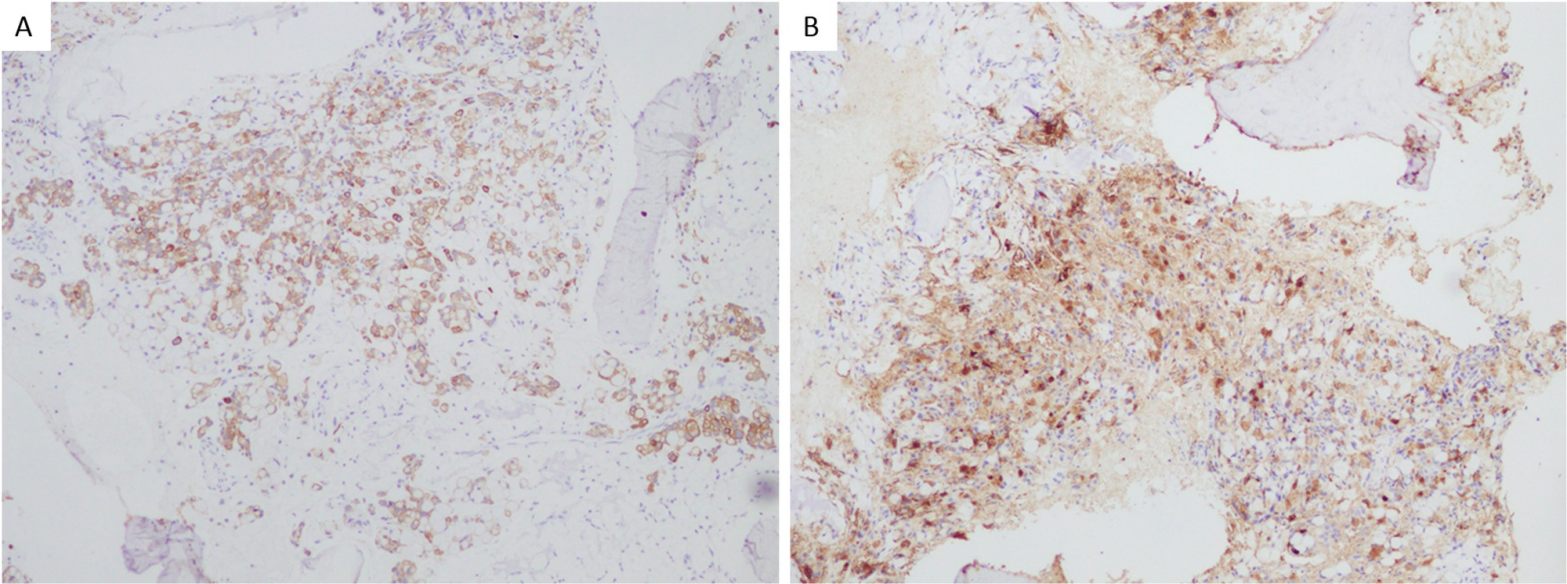
Immunohistochemistry on bone marrow biopsy showing positive staining of signet ring cells for CK7 and CDX2 (A) and CA 19-9 (B).

Further lab work revealed an iron level of 37 MCG/DL (normal range 65 - 175 MCG/DL), total iron-binding capacity 207 MCG/DL (normal range 250-450 MCG/DL), ferritin 1,003 NG/ML (26-388 NG/ML), and erythrocyte sedimentation rate (ESR) >120 mm/hr. The peripheral blood smear was unremarkable. Electrophoresis and Coombs test were negative. Bone marrow biopsy of the right iliac crest revealed metastatic signet ring cell adenocarcinoma consistent with gastrointestinal primary (Figure 2). Immunohistochemistry showed positive staining for CA 19-9, CK7, CK20, and CDX2, indicating a gastrointestinal origin (Figures 3A-3B), with human epidermal growth factor receptor 2 (HER2) non-amplified. Bronchoscopy, bronchoalveolar lavage (BAL), and biopsies taken during endobronchial ultrasound-guided transbronchial needle aspiration (EBUS-TBNA) of the subcarinal lymph node demonstrated adenocarcinoma, gastrointestinal primary. Carcinoembryonic antigen level was elevated at 672.3 NM/ML. After confirmed signet cell carcinoma diagnosis, the patient began the leucovorin, fluorouracil, and oxaliplatin (FOLFOX) regimen, which he successfully completed and was discharged home. He was unable to undergo genetic testing for CDH1 mutation given financial constraints.

Two months later, after four cycles of FOLFOX chemotherapy, he presented to the emergency department for left lower quadrant abdominal pain, diarrhea, fevers, and tachycardia. He was found to have Salmonella bacteremia and subsequently treated with ceftriaxone. A repeat CT of the chest, abdomen, and pelvis showed the interval development of numerous hepatic lesions, stable abdominopelvic lymphadenopathy, with suspicious gastric mucosal wall thickening. He continued to have pain refractory to medication management. After extensive discussion with the patient and family, he was discharged home with hospice.

## Discussion

Gastric cancer was once the leading cause of cancer deaths worldwide, until the 1980s. Today, gastric cancer is the 15th leading cause of death in the United States with an estimated 11,140 deaths nationwide in 2019 [2]. The incidence of gastric cancer varies across the globe with the highest rates in South America, Eastern Asia, and Eastern Europe and the lowest rates in North America. The recognition and eradication of certain risk factors, such as H. pylori and other dietary and environmental risks, have contributed to the decline in the incidence of gastric cancer.

There are two types of gastric cancer: diffuse, or signet ring cell adenocarcinoma, and intestinal. Intestinal gastric cancer is more common in males and older age groups, whereas diffuse type is more common with younger age, as characterized by our patient, and carries a worse prognosis. In regards to diffuse type, it has been thought that signet ring histology is an independent predictor of a worse prognosis, however, an increasing number of studies now suggests that while it does not carry a worse prognosis, signet ring histology is associated with a more advanced stage of disease at presentation [3-5]. Traditional risk factors for intestinal gastric cancer, such as long-standing gastritis caused by H. pylori infection, do not play a role in the development of diffuse-type gastric cancer. Tissue diagnosis to confirm gastric cancer is best obtained by upper gastrointestinal endoscopy with biopsies of any suspicious appearing gastric lesions. To note, gastric mucosa may appear normal in patients with linitis plastica, as the tumor spreads within the submucosa, resulting in the potential for false-negative superficial mucosa biopsies.

The most common presenting symptoms of gastric cancer at initial diagnosis are weight loss and abdominal pain. Bone pain as an initial presentation of metastatic signet ring cell adenocarcinoma has been rarely documented [6-7]. If bone metastasis is detected, it is typically during a metastatic workup and if it occurs, it is more commonly associated with signet ring cell adenocarcinoma [8]. Metastatic signet-ring cell gastric cancer is a highly aggressive malignancy and unlike typical types of gastric cancer, it does not follow the typical sites of metastatic involvement. This case report is significant, as it not only highlights a common complaint of back pain as the initial manifestation of gastric cancer, but it also shows a diagnosis of metastatic signet ring cell adenocarcinoma confirmed on bone marrow biopsy, which was not detected via endoscopy.

## Conclusions

While metastasis to the bone does occur with gastric cancer, extensive bone marrow infiltration as the initial presentation without preceding gastrointestinal symptoms is rare. This case highlights the importance of maintaining a broad differential in patients who present with bone pain and findings of lytic bone lesions. Given that our patient’s bone marrow biopsy results were consistent with gastric adenocarcinoma primary despite negative upper endoscopy biopsies and no gastric wall thickening or poor distention noted on imaging, this suggests the importance of incorporating a thorough workup when evaluating future patients for gastric malignancy. Bone marrow metastases as an initial presentation of gastric cancer will likely play a larger role in the diagnosis of gastric cancer, especially in younger patients in whom the cancer can be quite aggressive with an overall poor prognosis.
